# Dietary supply and reporting reliability during Antarctic overwintering under extreme isolation: a food image-based analysis

**DOI:** 10.3389/fnut.2026.1855383

**Published:** 2026-08-19

**Authors:** Verena Steinhauser, Bea Klos, Rebecca Föhr, Sophia Wolf, Eva Dichiser, Julia Low, Christine Brombach, Nadja Albertsen, Stijn Thoolen, Hannes Hagson, Paul Enck, Isabelle Mack

**Affiliations:** 1 Internal Medicine VI, Psychosomatic Medicine and Psychotherapy, University Hospital Tübingen, Tübingen, Germany; 2 Sensory and Consumer Science Research Group, School of Science, Royal Melbourne Institute of Technology (RMIT) University, Bundoora, VIC, Australia; 3 Research Group for Food Perception, Zurich University of Applied Sciences (ZHAW) School of Life Sciences and Facility Management, Waedenswil, Switzerland; 4 French Polar Institute Paul-Emile Victor, Brest, France - sponsored by the European Space Agency, Brest, France; 5 Aarhus University, Aarhus, Denmark; 6 Aarhus University Hospital, Aarhus, Denmark; 7 Department of Psychiatry, Massachusetts General Hospital, Harvard Medical School, Boston, MA, United States

**Keywords:** Antarctica, Concordia Station, diet, food image analysis, isolated confined extreme environment, sensory quality

## Abstract

**Background:**

Objective data on the actual food environment in isolated, confined, and extreme (ICE) settings remain scarce, as planned menus often differ from meals served. This study used image-based documentation to characterize meal composition and sensory properties during Antarctic overwintering and to assess agreement between planned and served meals.

**Methods:**

More than 2,000 photographs of lunch and dinner plates self-selected by the research physicians at Concordia Station, Antarctica, during three overwintering missions (Mar-Oct) were analyzed. Meals were classified by food group, color, and texture. Planned menus were compared with photographed meals to evaluate agreement. Because documentation varied across missions, analyses were descriptive.

**Results:**

Meal provision showed limited variation across campaigns and overwintering phases despite differences in personnel and documentation practices. Core food groups consistently dominated meal composition and were photographed on most days, with minor shifts across overwintering phases. Fresh and highly perishable foods declined early and remained minimal during winter, while shelf-stable foods showed broadly similar contributions. Visual appearance and texture profiles showed limited variation on descriptive inspection, indicating a relatively consistent food environment under prolonged isolation. Agreement between planned menus and photographed meals was only moderate (25–47%).

**Conclusion:**

Antarctic overwintering is characterized by a broadly structured and relatively consistent food environment that persists despite logistical constraints and prolonged isolation. Discrepancies between planned and served meals emphasize the need for objective documentation methods. These insights are relevant for designing and monitoring nutrition systems in other long-duration, confined environments, where image-based approaches may provide a complementary and scalable tool with reduced participant burden and improved ecological validity.

## 1 Introduction

Long-term residence in isolated, confined, and extreme (ICE) environments such as Antarctic research stations requires individuals to rely almost entirely on a fixed and pre-planned food supply for extended periods of time. Limited resupply opportunities and restricted availability of resources shape everyday life in these settings, particularly during periods of prolonged isolation such as the Antarctic winter (1–3). Daily routines, environmental exposures, and available resources are therefore tightly structured (3–5). Under these conditions, food is not only a source of nutrients but also a central and repeatedly encountered element of the immediate environment.

Within these environments, nutrition plays a central role in maintaining health and functional capacity because adequate energy and nutrient intake support energy balance and body mass regulation as well as key physiological functions and psychological wellbeing (5–9). At the interpersonal level, shared meals are associated with greater social connectedness and may act as social anchors where opportunities for interaction are limited (10, 11), as scheduled meal times may represent one of the few regular occasions for crew members to meet and interact.

Evidence from ICE missions such as spaceflight and Antarctic expeditions indicates considerable variability in dietary intake under ICE conditions, often accompanied by low energy intake and restricted consumption of fresh foods such as fruits and vegetables (2, 12, 13). At the same time, food frequency data suggest that shifts at the food-group level under constrained supply do not necessarily translate into major changes in macronutrient distribution (13), with protein typically showing less variability than fat and carbohydrate (14). This combination of variable intake but relatively stable macronutrient distribution may reflect structural constraints of the food environment, but could also indicate regulatory processes through which individuals maintain a preferred macronutrient balance despite constrained supply.

Whether such regulatory processes are further shaped by the sensory properties of available foods, however, remains unclear. Sensory characteristics such as color, variety, and texture influence expectations, perceived palatability, and intake (15–17), and may be particularly relevant in isolated environments where dietary variety is inherently constrained and food constitutes one of the few consistent sources of sensory stimulation in everyday life (16). Greater sensory variety is associated with higher acceptance and consumption, whereas monotony can lead to menu fatigue and declining intake over time (18, 19). Color serves as a primary visual cue for food quality and appeal (17), and has been proposed as a visual proxy for nutritional variety (20), with evidence suggesting that color variety can influence intake and food acceptance (21). Texture further modulates eating rate and energy intake (22, 23). Despite their potential relevance for supporting adequate food and energy intake, sensory characteristics of meals in ICE environments have rarely been examined systematically.

Most evidence on diet in ICE environments is derived from self-reported dietary assessments or planned menus (14). While informative, these approaches provide only indirect insight into what is actually available and served. Planned menus may differ from meals ultimately provided due to substitutions, shortages, or operational adaptations, and self-reports remain susceptible to recall bias and misreporting (24, 25). Objective, day-to-day information on the experienced food environment during isolation therefore remains limited, both with respect to nutritional composition and sensory characteristics.

Image-based methods may offer a promising approach to capture both compositional and sensory aspects of meals and enable the assessment of real-world food environments beyond self-report and menu-based measures. While such approaches have been applied to evaluate food environments and menu quality in other contexts (26), their use in Antarctic overwintering remains scarce.

To address this gap, the present study used systematic photographic documentation to characterize meals served during three overwintering campaigns at Concordia Station. The aims were (1) to describe meal composition and food group diversity of the research physicians' photographed plates as an indicator of food availability under specific ICE conditions, (2) to assess sensory aspects of meals, including color and texture, to capture visual diversity and (3) to examine agreement between planned menus and photographed meals, to evaluate the utility of image-based documentation as a method to characterize food environments under ICE conditions. By providing an objective, image-based perspective on the food environment, this study contributes to a more detailed understanding of dietary conditions in Antarctic ICE settings and may inform food provisioning strategies in other isolated and confined environments.

## 2 Methods

### 2.1 Study design

This study applied an observational, image-based design to characterize the meal composition of self-selected lunch and dinner plates, documented within the communal dining setting, under isolated and confined conditions at Concordia Station, Antarctica. Meal documentation formed part of routine medical and nutritional monitoring during multiple overwintering missions, with planned daily photographic documentation of officially organized lunch and dinner meals, supplemented by planned menu. Breakfast and between-meal food intake were not documented, as these eating occasions were highly individualized and, in the case of between-meal intake, not routinely practiced due to logistical constraints and limited access to food supplies. The analysis therefore focused on descriptive evaluation of the shared food environment based on photographic documentation and planned menus.

The study was carried out in accordance with the ethical principles of the Declaration of Helsinki. The study protocol was approved by the Ethics Committee of the University Hospital Tübingen, Germany (NCT03746145).

### 2.2 Study setting

The study was conducted at Concordia Station (Dome C, Antarctica), a remote research facility located on the Antarctic Plateau (75°06′ S, 123°23′ E) at an altitude of 3,233 m above sea level. Due to reduced barometric pressure, the physiological altitude corresponds to approximately 3,800 m, resulting in chronic exposure to hypobaric hypoxia. The station environment is characterized by extremely low temperatures, minimal humidity, and prolonged periods of darkness during the polar night, accompanied by prolonged isolation and limited environmental variation (3).

The station is jointly operated by the French Polar Institute (IPEV) and the Italian Antarctic Program (PNRA) and accommodates a multinational winter-over crew of 12–15 personnel. Each overwintering mission involved a distinct crew that remained at the station throughout the full winter period. During the 9-month isolation phase (February–November), the station operates under complete logistical autonomy, with no possibility of external resupply.

Food provision follows a predefined annual resupply cycle. Fresh fruits and vegetables are primarily available after the summer resupply (December–February), whereas frozen and canned foods are used during the remaining months.

This setting can be described as a semi-structured food environment combining centralized provisioning with individual-level choice. Breakfast and snacks are prepared individually, whereas lunch and dinner are centrally prepared on a daily basis by a station chef, occasionally incorporating leftovers or reused components from previous days. These meals typically consist of multiple components (e.g., starter, main course, dessert).

Meals are generally consumed in a shared dining setting at fixed times, although participation is not strictly mandatory. Food provision follows a structured format, while individual meal composition allows for some degree of choice, as crew members can select portion sizes and specific components (e.g., side dishes). All meals are consumed *ad libitum*.

Across overwintering campaigns, meal preparation was conducted by different station chefs (e.g., French and Italian backgrounds).

### 2.3 Data collection

#### 2.3.1 Photographic documentation

Meal photographs were collected during the 2018/2019, 2019/2020, and 2021/2022 overwintering missions at Concordia Station as part of routine medical and nutritional monitoring, with analyses restricted to the isolated overwintering phase from March to October to ensure comparable seasonal coverage across campaigns. No data were collected during the 2020/2021 season due to COVID-19-related operational restrictions.

In each mission, the station research physician (one female and two male physicians across missions) photographically documented their self-selected lunch and dinner meals served daily in the dining area. These meals were chosen from the foods available within the shared meal setting and represent individual selections from the centrally prepared menu. The resulting photographs therefore reflect the experienced food environment of the study physicians rather than the full range of foods offered or consumed by the entire crew.

To support comparability across sessions, images were acquired at a standardized angle of approximately 45°, at a consistent distance from the plate, under indoor artificial lighting conditions typical for the station dining area during the overwintering period. A reference coin of known diameter was placed next to each dish to support approximate portion size estimation, and the dimensions of commonly used plates were measured. Photographs were acquired using a compact digital camera (Canon IXUS 185, Canon Inc., Tokyo, Japan).

#### 2.3.2 Image analysis

Photographic records were prepared for analysis by uploading all images into a custom-developed image analysis tool (University Hospital of Tübingen, Germany), designed to enable structured annotation of meals based on predefined categories. The tool allowed standardized recording of food group proportions as well as visual characteristics including color and texture, and facilitated systematic comparison of meals across days and overwintering periods. Planned menus were integrated as supplementary information to support correct assignment and interpretation of meals. Images were organized chronologically by recording date, enabling day-by-day and meal-specific (lunch and dinner) assessments.

To ensure consistent evaluation, only complete meals were included. Meals were excluded if plates showed signs of prior consumption or if visible meal components were missing at the time of documentation. More than 2,000 images met inclusion criteria and were independently evaluated by two trained raters, who were trained using a standardized evaluation protocol prior to analysis. Representative example images illustrating the applied classification criteria for food group assignment, color coding, and texture classification are provided in Supplementary Figure S1.

Each image was evaluated by trained human raters along three predefined dimensions (Table 1): food group classification, color characteristics, and texture. These dimensions defined the manual assessment procedure and were applied consistently across all photographs. Image-processing tools were used only to support color extraction and visualization.

**Table 1:** Classification criteria used for image-based meal analysis.

Dimension	Definition/criteria
Dimension (i) Food group classification	
**Food group**	**Definition**
Vegetables	Cooked vegetables, salads and raw vegetables
Fruits	Fresh, cooked, or processed fruits
Cereals and cereal products	Pasta, rice, cereals, bread, and sweet or savory dough products
Potatoes	All potato preparations
Nuts	Whole or processed nuts
Meat and meat products	Red, white, and processed meat
Fish	Fish and seafood
Milk and milk products	Milk, yogurt, dairy desserts and all cheese types
Eggs	Eggs
Fats	Visible fats such as oils, spreads, and frying fats
Soups and Sauces	Liquid or semi-liquid dishes and accompaniments
Sweets	Sweets and confectionery
Dimension (ii) Color classification	
**Color group (HSL-based, adapted)**	**Hue range (°)**
Reds	0–15 and 345–360
Oranges	15–40
Yellows	40–55
Greens	55–135
Green-Cyans	135–165
Cyans	165–195
Cyan-Blues	195–225
Blues	225–255
Blue-Magentas	255–285
Magentas	285–315
Magenta-Reds	315–345
Dimension (iii) Texture classification	
**Level (according to IDDSI)**	**Description**
Level 3—Liquidized	Fully smooth and pourable
Level 4—Pureed	Smooth, thick, no chewing required
Level 5—Minced and moist	Small soft pieces, minimal chewing
Level 6—Soft and bite-sized	Tender, chewable pieces
Level 7—Regular	Normal textures requiring chewing

Image-based meal classification framework using food groups adapted from the European Prospective Investigation into Cancer and Nutrition (EPIC), color categories derived from Hue–Saturation–Lightness (HSL) values with minor adaptations of hue ranges for food classification, and texture levels according to the International Dysphagia Diet Standardization Initiative (IDDSI).

Food groups were categorized using a scheme adapted from the European Prospective Investigation into Cancer and Nutrition (EPIC) (27, 28). For each identified food category, raters estimated its proportional contribution to the total plate content. When multiple plates were presented within a single meal, proportions were estimated across all visible items. In addition to proportional estimates, frequencies of occurrence (% of observation days) were calculated. Visible fats represented an exception, as their quantities were not reliably estimable from images; these were therefore recorded as binary presence/absence variables and included only in frequency analyses.

Color assessment was based on characteristic pixels selected per food item within each photograph, rather than on whole-image or region-based pixel segmentation. The hexadecimal color value of this pixel was converted to Hue-Saturation-Lightness (HSL) values, and the hue angle was assigned to one of the predefined color categories (Table 1). Reported percentages therefore reflect the proportion of documented food items assigned to each color category. Color variety was quantified as the number of distinct color points per meal, and perceived color impression was rated on a three-point scale (dull, moderate, colorful). No color calibration standards were available, and automated white-balance normalization could not be reliably applied owing to the substantial shadow and neutral-gray areas present in the meal images. Given this exploratory approach, the resulting analyses are suited to broad, descriptive comparisons rather than precise colorimetric measurement.

Texture was visually classified according to the International Dysphagia Diet Standardization Initiative (IDDSI) framework for food (levels 3–7) (29, 30), based on visible consistency characteristics in the photographs. The IDDSI framework was selected as a standardized and internationally recognized reference system for describing food texture and thickness, enabling reproducible classification from image-based assessments, although the resulting classifications reflect visual approximations rather than validated physical measurements.

To derive the final dataset, continuous ratings from both raters were averaged, while categorical discrepancies were resolved through joint review to reach consensus. Interrater reliability was assessed across all three coding dimensions, including food group proportions, color characteristics, and texture classification. Overall agreement was moderate to excellent across most categories, with Intraclass Correlation Coefficient (ICC) values ranging from 0.687 to 0.976 for food groups, from 0.979 to 0.999 for colors, from 0.646 to 0.849 for color dots, and from 0.914 to 0.979 for textures (all *p* < 0.001). Cohen's Kappa values for the binary-coded food group categories were similarly moderate to excellent for most categories (0.602–0.978), with lower agreement observed only for select visible-fat subcategories, specifically oil (Kappa 0.375) and spread (Kappa 0.179; all *p* < 0.001).

#### 2.3.3 Cook records

As an additional documentation source, daily planned menu logs were compared with photographic records to assess concordance between menu records and the meals photographed by the research physicians. Because planned menus did not consistently reflect the meals photographed, they were not used for coding decisions. Instead, comparisons were conducted separately at the dish level to quantify agreement between planned and documented meals; for meals comprising multiple courses (e.g., starter, main course, dessert), each dish was evaluated separately against the corresponding menu entry, and side dishes were included in the comparison. A match was defined as identical or equivalent dishes recorded in both sources. Equivalent dishes were defined as meals sharing the same primary ingredient or core dish category (e.g., beef-based main dishes or pasta dishes), despite minor differences in preparation method, seasoning, or presentation. Partial matches, defined as dishes sharing some but not all components with the corresponding menu entry, were classified as non-matches. Agreement percentages were calculated as the number of complete matches divided by the total number of dishes compared within each campaign. Menu logs were used solely for consistency checks and did not allow reconstruction of the full range of foods offered to the entire crew at each meal.

#### 2.3.4 Statistical analysis

Statistical analyses were performed using IBM SPSS Statistics version 28 (IBM Corp., Armonk, NY, USA). Graphical visualizations were prepared using GraphPad Prism version 10.1.1 (GraphPad Software, San Diego, CA, USA).

Analyses followed a descriptive and exploratory approach. No inferential statistical tests were conducted. Although standardized documentation procedures were defined, data collection occurred under routine station conditions and showed variability in documentation completeness and recording practices across missions and personnel. Furthermore, although the dataset spans three overwintering campaigns, it does not constitute a repeated-measures design in the statistical sense: each campaign was conducted by different personnel with varying documentation completeness, and no within-individual longitudinal tracking across missions was performed. These structural differences mean that the assumptions underlying mixed-effects models or time-series analyses, including consistent measurement conditions and comparable units of observation across time points, were not met. Analyses were therefore restricted to descriptive statistics. Stability in meal composition and sensory characteristics was assessed by examining the range and distribution of proportional contributions across campaigns and time periods; patterns were considered broadly consistent when visual inspection indicated limited fluctuation without a systematic directional trend.

Proportional data for food groups, color categories, and texture levels were aggregated at the meal level and summarized by month and by overwintering period. Frequencies of occurrence were calculated as the percentage of observation days with the presence of a given category. Descriptive statistics are presented as means ± standard deviations (SD) for continuous variables and as percentages for categorical variables.

Interrater reliability was evaluated using a two-way mixed-effects, consistency, average-measures ICC model [ICC(3,k)] for proportional data (31) and Cohen's kappa (32) for binary variables, as described in the image analysis section.

## 3 Results

### 3.1 General overview

Across the three overwintering periods (2018/2019, 2019/2020, 2021/2022), 2,066 food images from 593 days were available, of which 549 days fell within the predefined analysis window (March 1–October 31). Within this period, 1,098 main courses, 364 starters, and 184 desserts were documented photographically (Table 2).

**Table 2:** Overview of photographic documentation and analysis inclusion by overwintering campaign.

Dimension	2018/2019	2019/2020	2021/2022	Total
Overwintering period (full duration)	Feb 12–Nov 6, 2019 (268 days)	Feb 7–Nov 12, 2020 (280 days)	Feb 7–Nov 6, 2022 (273 days)	–
Days with photographic documentation, n/total days in overwintering period, *n* (%)	90/268 (34%)	262/280 (94%)	241/273 (88%)	593
Days with photographic documentation within analysis window (Mar 1–Oct 31), *n*/analysis window (245 days) (%)	84/245 (34%)	227/245 (93%)	238/245 (97%)	549
Main courses documented within analysis window, *n*	168	454	476	1,098
Starters documented within analysis window, *n*	116	145	103	364
Desserts documented within analysis window, *n*	6	147	31	184

“Days with photographic documentation” refers to all days on which at least one meal photograph was taken. The analysis window (March 1–October 31) was applied to ensure comparable seasonal coverage across campaigns (245 days per campaign). Percentages are rounded to the nearest whole percent.

Photographic coverage varied between campaigns, ranging from partial documentation in 2018/2019 (84/245 days) to near-complete coverage in 2019/2020 (227/245 days) and 2021/2022 (238/245 days). These differences reflect variations in documentation practices and operational routines across campaigns and should be considered when interpreting between-campaign comparisons. Although completeness varied, the resulting dataset provided sufficient temporal coverage to capture recurring food provision patterns and support pooled descriptive analyses.

Meals were prepared by different station cooks in each campaign (1 woman, French, and 2 men, both Italian) and photographed by the station research medical doctors (1 woman, 2 men). Differences in culinary background, food preferences, and handling of side dishes or desserts may have contributed to some between-campaign variability.

Meals typically consisted of a carbohydrate-based main dish, one protein source (meat or fish), and side dishes such as vegetables or starches, suggesting a broadly consistent meal structure during overwintering as documented in the photographic records.

**Figure 1:**
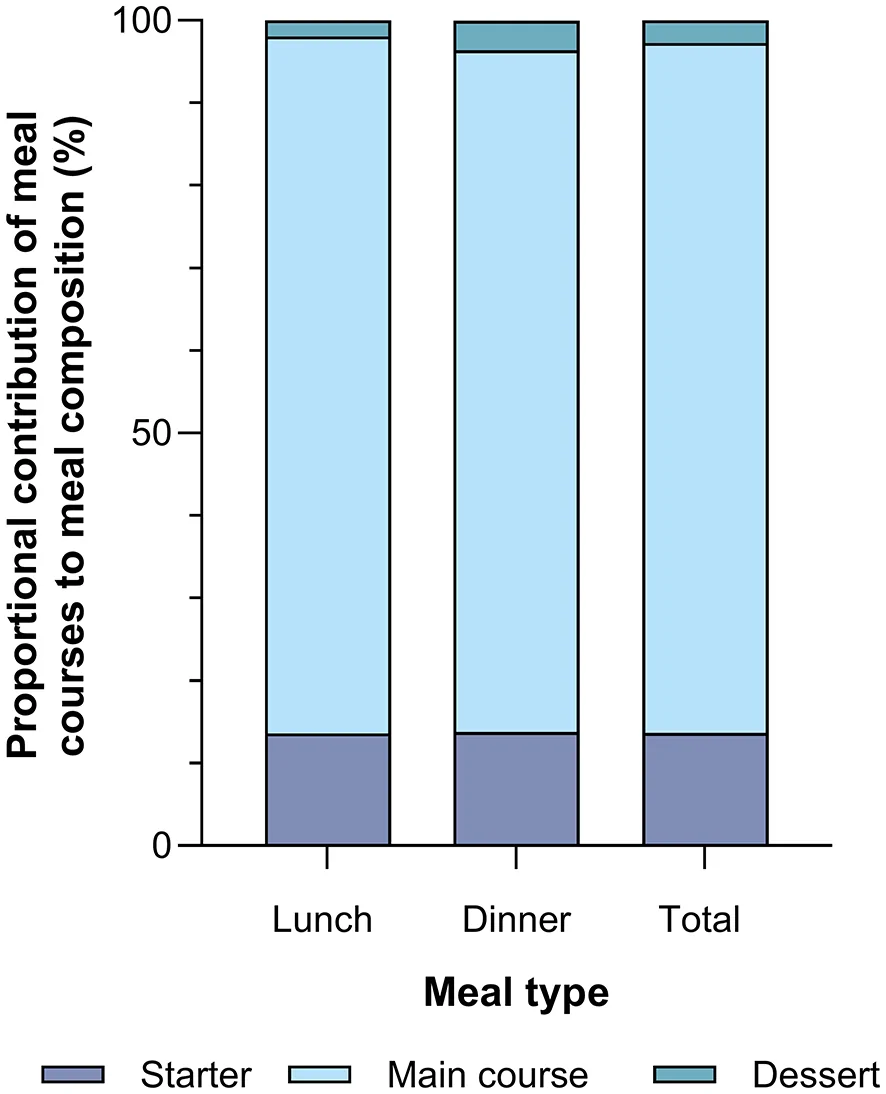
Proportional contribution of meal courses by meal type. Stacked bars show the mean proportional contribution (%) of starter, main course, and dessert to the whole meal, presented separately for lunch, dinner, and overall totals. Proportions are expressed relative to the total meal composition per recorded meal, allowing comparison of course distribution by meal type.

Photographic records consistently showed a course structure dominated by the main course (Figure 1). When pooled across campaigns, the main course accounted for the majority of meals at both lunch and dinner. Starters contributed a secondary share, whereas desserts represented only a minor proportion. This pattern was observed in all campaigns despite differences in documentation intensity (Supplementary Figure S2).

Because of variation in photographic coverage, personnel, and documentation practices, formal statistical comparisons between overwintering campaigns were not performed. Descriptive patterns, however, indicated broadly comparable meal structures and provisioning across years, though heterogeneity in documentation completeness should be considered when interpreting this comparison.

#### 3.1.1 Core food groups recurrently shaped meal composition, suggesting a broadly structured provisioning pattern with limited fluctuation across campaigns

**Figure 2:**
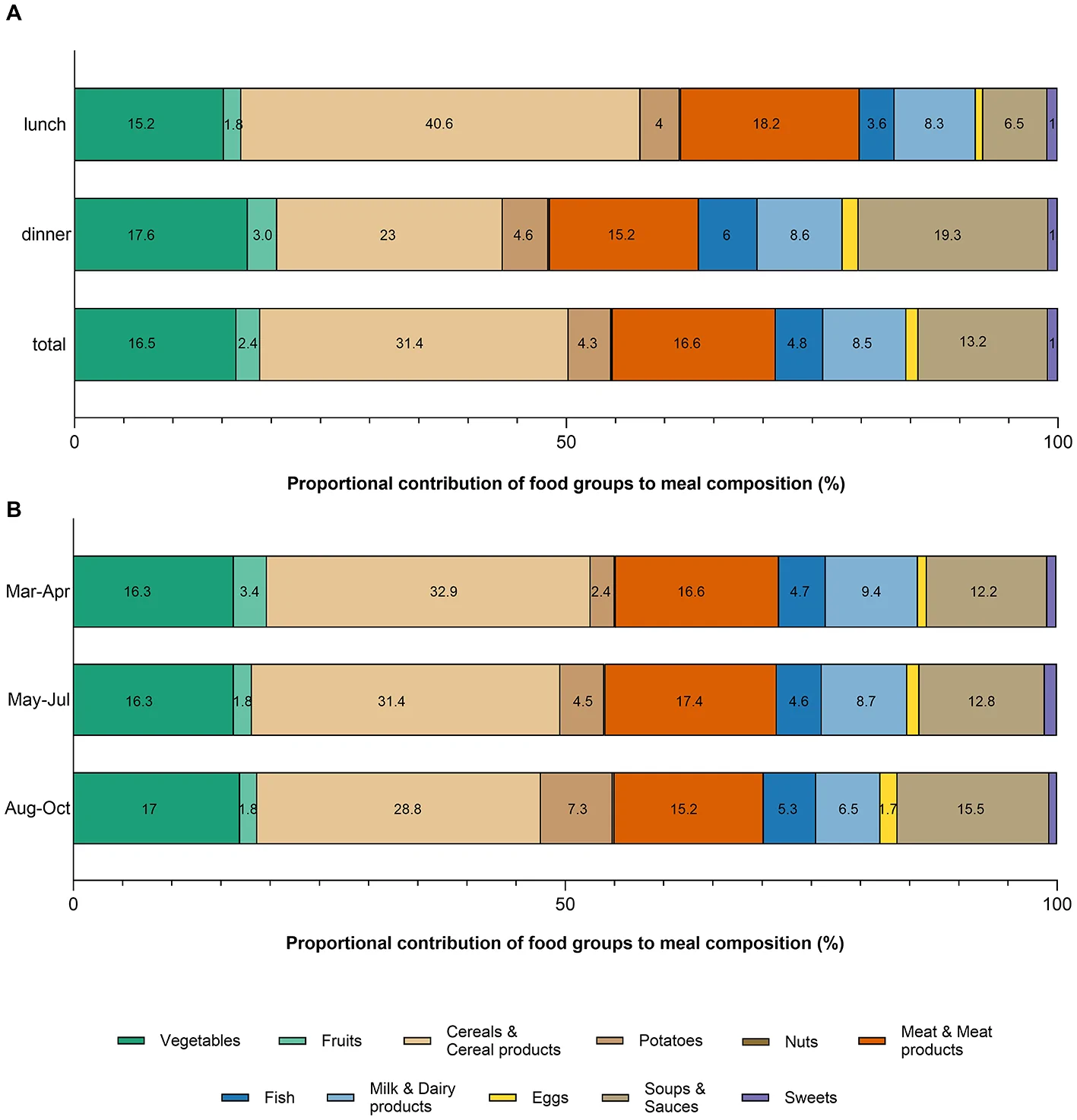
Proportional contribution of food groups to meals by meal type and time period. **(A)** Stacked bars show the mean proportional contribution (%) of individual food groups to the whole meal presented separately for lunch, dinner, and overall totals. **(B)** Stacked bars show the mean proportional contribution (%) of individual food groups to the whole meal by time period, defined as early (March–April), mid (May–July), and late (August–October) phases of the overwintering mission. Values are expressed as percentages of total meal composition, allowing comparison of relative food group contributions by meal type and across time periods.

Across overwintering campaigns, meal composition was predominantly characterized by cereals and cereal products, vegetables, and meat and meat products, which together accounted for the largest share of total meal composition (Figure 2; Supplementary Figure S3). Cereals and cereal products formed the principal component throughout, typically contributing about one quarter to one third of total meal composition, while vegetables (≈14–28%) and meat and meat products (≈15–26%) also represented substantial shares.

Meal-type comparisons showed similar overall patterns for lunch and dinner (Figure 2A). Cereals contributed more strongly at lunch (lunch 41% vs. dinner 23%), whereas soups and sauces (lunch 7% vs. dinner 19%) and fish (lunch 4% vs. dinner 6%) appeared more frequently at dinner, without altering the dominance of the core food groups.

Across overwintering phases, food group composition showed limited variation across early to mid (Antarctic winter) and late mission phases (Figure 2B; Supplementary Figure S4). Fresh products, particularly salads and raw vegetables, declined sharply early in each overwintering period and contributed < 1% during most winter months. Shelf-stable or frozen foods showed limited variation across phases.

**Figure 3:**
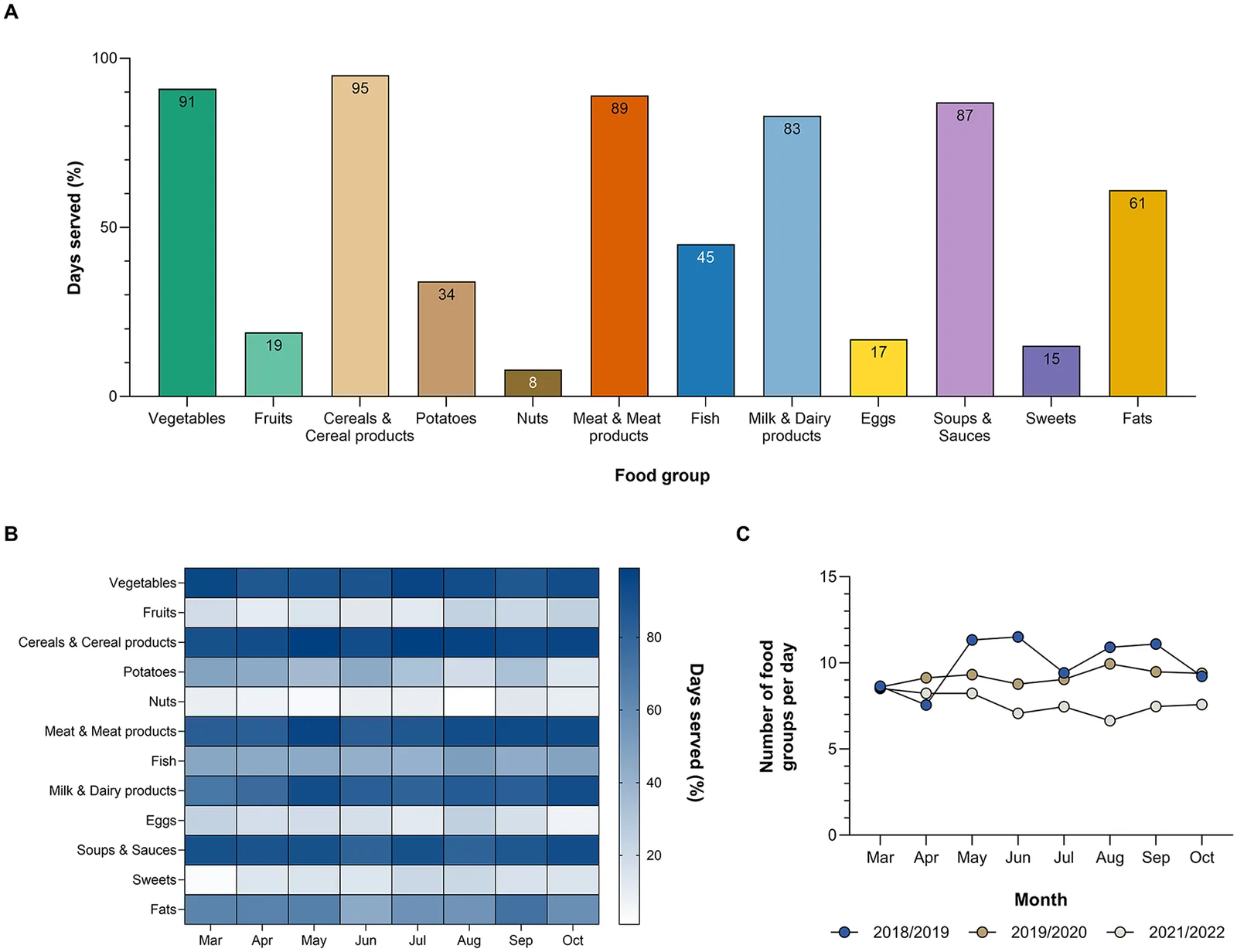
Frequency of food groups served. **(A)** Frequency of food groups. Bars show the mean percentage of recorded days on which each food group was served. **(B)** Frequency of food groups by month. Heatmaps display the mean percentage of recorded days each food group was served, summarized by month, allowing comparison of temporal patterns in food group provision. **(C)** Number of food groups by overwintering campaign. Points show the average number of food groups served per day (mean): 2018/2019 (blue), 2019/2020 (tan), and 2021/2022 (light gray), allowing comparison across campaigns.

To assess whether this descriptively observed consistency reflected a structured pattern rather than incidental variation, the occurrence of food groups in the photographed plates was examined as an indicator of food availability under specific ICE conditions. The same core food groups dominating meal composition were also served on most days, typically on >80–90% of recorded days (Figure 3A; Supplementary Figure S5). Descriptively, fluctuations appeared minimal and no systematic seasonal pattern was apparent (Figure 3B; Supplementary Figure S6). Daily dietary variety averaged 8–10 food groups and showed limited variation across campaigns on descriptive inspection (Figure 3C).

#### 3.1.2 Food provision showed limited variation in visual meal appearance over time

**Figure 4:**
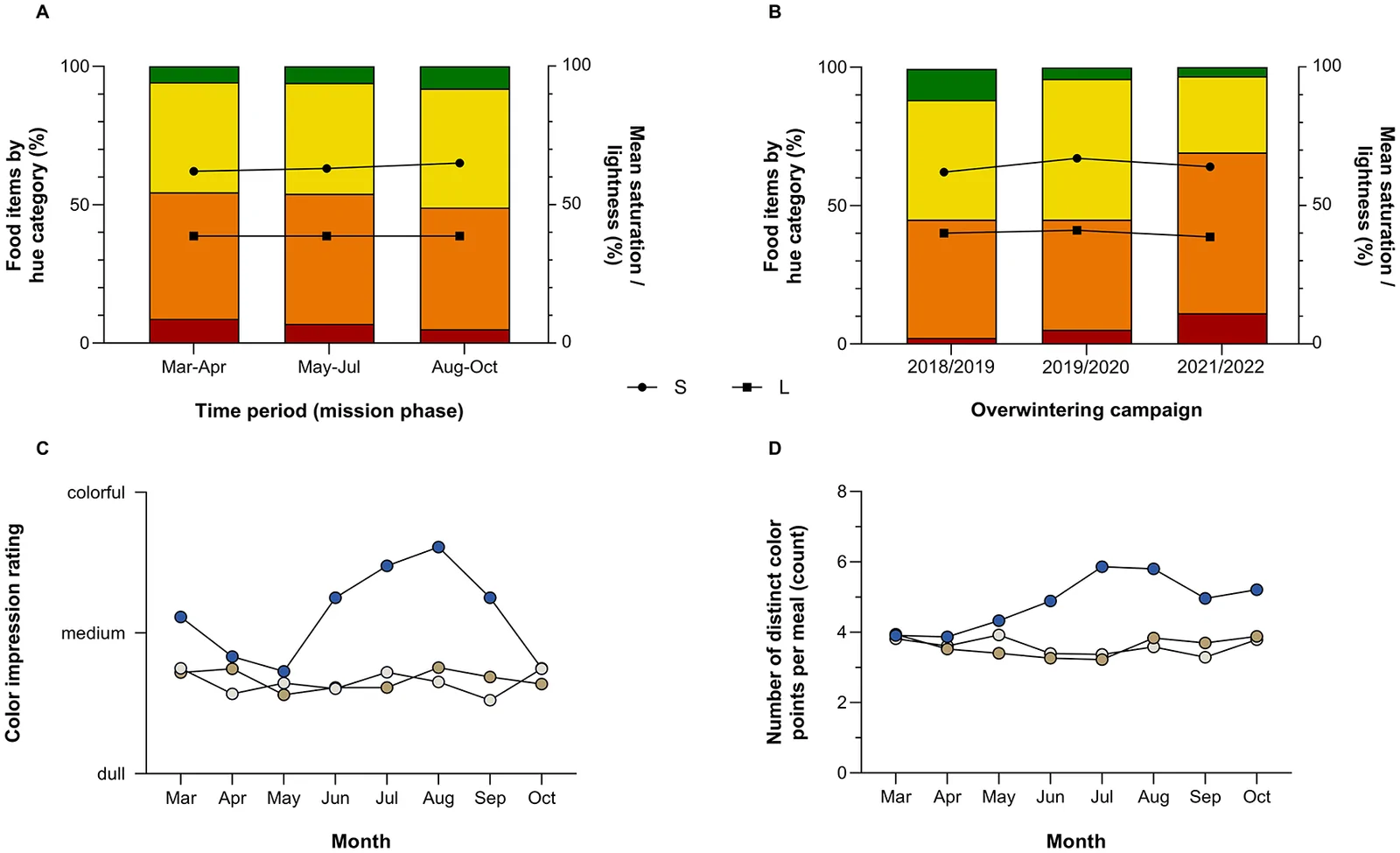
Color characteristics of meals across time periods and campaigns. **(A)** Color classification by time period. Stacked bars show the mean percentage of documented food items assigned to each color category, based on the hue value of characteristic pixels selected per food item and converted from hexadecimal to HSL values. Time periods represent phases of the overwintering mission—early (March–April), mid (May–July), and late (August–October). Color classification was performed in the HSL color space using predefined Hue (H) angle ranges (see Table 1). Overlaid symbols indicate mean Saturation (S; circles) and Lightness (L; squares) values by time period. Saturation reflects chromatic intensity or color purity (lower values indicating more desaturated or grayish tones, higher values more vivid colors), whereas Lightness represents perceived brightness from dark to light. **(B)** Color classification by overwintering campaign. Stacked bars show the mean percentage of documented food items assigned to hue-based color classes for overwintering campaigns 2018/2019, 2019/2020, and 2021/2022, allowing comparison of overall color composition between campaigns. Color classification was performed in the HSL color space using predefined Hue (H) angle ranges (see Table 1). Overlaid symbols indicate mean Saturation (S; circles) and Lightness (L; squares) values by overwintering campaigns. **(C)** Color impression by month. Points show the mean color impression rating per meal by month, averaged across two independent raters, based on a three-level scale (1 = dull, 2 = moderate, 3 = colorful), allowing comparison of perceived meal colorfulness across months. **(D)** Number of color points by month. Points show the mean number of distinct color points per meal by month, averaged across two independent raters, reflecting the variety of visible colors within meals.

Beyond what was served and how often, the sensory characteristics of meals provide insight into how the food environment was visually experienced during prolonged isolation. Across overwintering, visual meal appearance appeared relatively uniform over time on descriptive inspection, without systematic temporal trends. Meals were predominantly characterized by yellow and orange tones across early, mid (polar night), and late mission phases, whereas green and red contributed only small proportions. Color saturation and lightness followed a similar pattern, exhibiting only minor fluctuations across time periods (Figure 4A; Supplementary Figure S7). When comparing campaigns, between-campaign differences appeared more pronounced than temporal changes within campaigns on descriptive inspection (Figure 4B). The 2018/2019 and 2019/2020 campaigns displayed relatively balanced contributions of yellow and orange hues, whereas 2021/2022 showed a stronger predominance of orange and red tones and lower contributions of other hues.

Subjective ratings from the trained graders reflected broadly similar patterns (Figures 4C, D). Meals were most often rated as dull to moderately colorful, with the number of distinct color points per meal typically ranging from 3 to 5. While variability across months was moderate, no consistent temporal trend emerged within campaigns. However, both color impression scores and color point counts were higher overall in 2018/2019 than in 2019/2020 or 2021/2022, and in 2018/2019, both measures showed a gradual increase from early to late overwintering phases.

While color describes visual aspects of meals, texture reflects their structural and oral properties and thus represents another sensory dimension of the eating environment.

**Figure 5:**
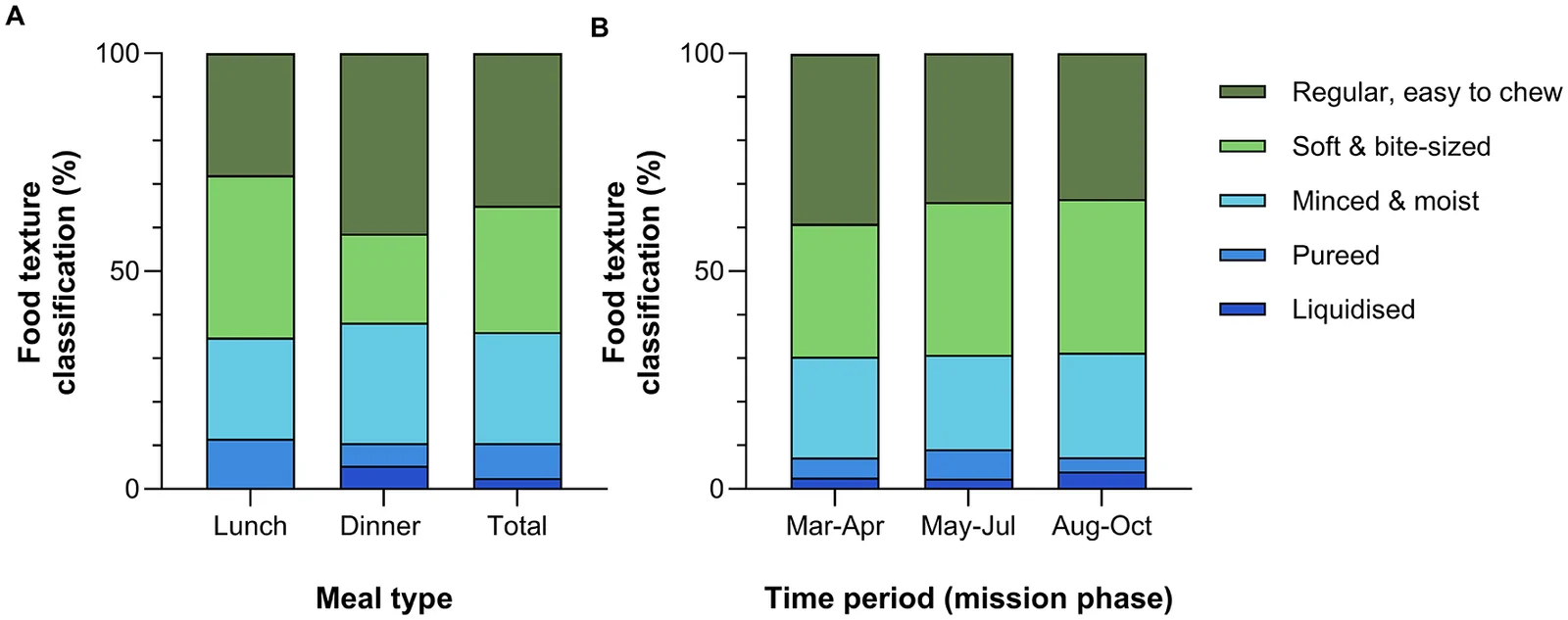
Distribution of food textures by meal type and time period. Mean percentages of food textures classified according to the International Dysphagia Diet Standardization Initiative (IDDSI) framework. Bars represent percentages, allowing comparison of texture distributions. IDDSI levels describe standardized texture and thickness characteristics (see Table 1), with lower levels indicating thinner, more liquid consistencies and higher levels representing increasingly solid textures. **(A)** Comparison of texture distributions between lunch and dinner meals and totals across all overwintering campaigns. **(B)** Comparison of texture distributions across timepoints during the overwintering period across all overwintering campaigns.

Across overwintering campaigns, meal texture profiles showed descriptively a high degree of consistency. Meals were predominantly characterized by soft to moderately firm textures, with liquid, creamy, mushy, tender, and al dente components occurring regularly, whereas firm or crunchy textures were less frequent. Within the IDDSI framework, these consistencies corresponded mainly to soft/bite-sized and regular textures (levels 6–7), with smaller but consistent contributions of softer, spoonable textures (levels 3–5) (Figure 5).

Descriptively, temporal variation within campaigns appeared limited. Although minor shifts were observed, such as a gradual increase in liquid textures during the 2019/2020 campaign (6% at the beginning vs. 10% at the end of overwintering), overall texture profiles showed no marked systematic shifts over time.

Between-campaign differences appeared modest (Supplementary Figure S8). Although soft/regular textures predominated throughout, 2018/2019 showed a comparatively higher proportion of regular textures (48% vs. 32% in 2019/2020 and 24% in 2021/2022), whereas 2021/2022 exhibited a relatively higher proportion of softer consistencies (36% vs. 24% in 2018/2019 and 27% in 2019/2020). Despite these differences, overall texture distributions appeared broadly comparable across campaigns on descriptive inspection.

#### 3.1.3 Photographic documentation indicated frequent deviations from planned menus

Agreement between planned menus and photographic documentation was limited, indicating that planned menus only partially reflected the meals actually served. Complete agreement between menu plans and photographed meals was observed in 30% (*n* = 47/158 matches) of cases in 2018/2019, 47% (*n* = 208/441 matches) in 2019/2020, and 25% (*n* = 114/457 matches) in 2021/2022. A complete match required that the dishes listed in the menu plan corresponded to the foods visible in the images. Discrepancies mainly reflected undocumented substitutions, spontaneous modifications, or differences in side dishes.

## 4 Discussion

The image-based analysis provides an objective description of the self-selected lunch and dinner plates of the research physicians during three Antarctic overwintering campaigns, offering a rare window into their experienced food environment under ICE conditions. As these plates were selected from the same centrally prepared menu shared with the full crew, they likely approximate what was more broadly available, though they cannot capture the full range of foods offered to or consumed by the entire crew. This food-system perspective, based on photographed plates rather than self-reports or planned menus, extends existing Antarctic nutrition research by capturing what was actually plated for the study physicians; an approach of particular value in operational settings where deviations from planned menus are common. Across campaigns, meal provision appeared structured and operationally robust, suggesting that consistent food provision may be maintainable even under severe supply constraints (3, 33). The findings are discussed in relation to the three study aims: meal composition and food group diversity, sensory characteristics including color and texture, and agreement between planned menus and photographed meals.

First, the results provide a characterization of meal composition and food group diversity across overwintering campaigns. Because maintaining energy and nutrient supply is a primary concern under ICE conditions (13, 34), the composition of the documented provision is directly relevant. Cereals and cereal products consistently formed the backbone of meals, mirroring reports from Antarctic and spaceflight contexts (8, 12) and reflecting their role as reliable energy sources given the demands of cold exposure, workload, and hypobaric hypoxia at Concordia (3). More broadly, seasonal influences on dietary intake have also been documented in temperate populations, with reported differences in food and nutrient intake between winter and summer and environmentally driven variation in energy intake (35, 36). Protein provision relied largely on meat and meat products, which are logistically advantageous due to storability and provide high-quality protein (37, 38). Although high habitual intakes of red and processed meat are associated with long-term health risks (39), Antarctic missions represent time-limited exposures, and prior data suggest that elevated meat consumption during overwintering tends to normalize after return (13). The low representation of fish, eggs, and dairy points to opportunities to diversify protein and calcium sources within existing logistical constraints; while sustainability-oriented dietary concepts are increasingly discussed (40, 41), shelf-stable formats of these foods may offer pragmatic options in ICE environments where energy adequacy and digestibility remain primary concerns.

Beyond macronutrient provision, the composition of side dishes offers insight into how nutritional quality and energy considerations are balanced under ICE conditions. While restricted access to fresh foods is often assumed to lead to declining dietary quality in Antarctica (1), the photographed meals suggest a more nuanced picture: salads and raw vegetables declined early in each campaign, yet the overall proportional contribution of vegetables showed limited decline on descriptive inspection. One plausible explanation for this pattern could be the regular provision of frozen or canned products, although this cannot be directly derived from the present data, as the classification framework did not differentiate by preservation method. As vegetables are among the foods most sensitive to declines in availability and sensory appeal (42), their presence on the plate represents a prerequisite for intake (43), and sustaining vegetable exposure remains relevant given their contribution to fiber, micronutrients, and gut and immune health (44–48). At the same time, in contexts where negative energy balance is a concern (14), promoting large amounts of low-energy-density foods requires careful consideration, as high-volume foods may promote early satiation before sufficient energy intake is achieved (49, 50). The present findings suggest that moderate vegetable provision may be feasible without displacing energy-dense foods, although this interpretation should be considered in light of the observational nature of the data and the absence of direct nutrient intake measurements.

As the present study assessed meal provision rather than actual intake, and breakfast and snacks were not captured, no conclusions can be drawn about individual energy consumption or adequacy (51). Nevertheless, the observed balance between carbohydrates, protein sources, and side dishes documented in the photographed meals suggests a broadly structured provision pattern, and adjustments in energy density through fat-containing or energy-dense accompaniments could represent feasible intervention points where higher intake is required (52).

Within this environmental framework, dietary monotony represents a known risk in isolated settings and has been linked to reduced appetite and intake (2, 53). The descriptively consistent number of food groups photographed per day suggests that structured provisioning can help limit monotony-related risks even when ingredient diversity is inherently limited. In ICE environments, where alternative sources of stimulation are scarce, food variety may carry disproportionate psychological relevance. Shared mealtimes may represent one of the few regular occasions for communal gathering, potentially supporting social cohesion and mood regulation among crew members (5, 54), and meals serve social and emotional functions that extend beyond nutritional adequacy (5, 11, 55). The compositional and sensory characteristics documented in the present study may therefore carry relevance for both nutritional and psychosocial dimensions of the food environment.

Second, the results provide insight into sensory characteristics of meals, particularly visual appearance and texture. Meals were visually dominated by yellow and orange hues and were frequently perceived as dull to moderately colorful, likely reflecting the predominance of cooked and starch-based components. Minor variation in dominant hues was observed between campaigns, but no systematic shifts in overall visual meal appearance were apparent on descriptive inspection. One campaign showed descriptively higher color diversity compared with the others, suggesting that greater visual variety is feasible within the same supply system; possible explanations may include differences in meal preparation practices, cook preferences, or variation in the use of preserved foods, though these factors were not systematically assessed. Between-campaign color comparisons should, however, be interpreted with particular caution, as no color calibration was applied; absolute hue values still reflect the specific capture conditions and cannot be regarded as standardized color measurements. Irrespective of absolute color values, limited color variety may reinforce perceptions of monotony: greater color variety has been shown to enhance intake and acceptance (21), whereas repeated exposure to similar sensory properties may promote sensory-specific satiety, reducing the desire for those foods (56). Texture profiles showed a descriptively similar degree of homogeneity, with soft to moderately firm consistencies predominating. Texture influences oral processing, eating rate, and energy intake (22, 23), and limited textural contrast may further reinforce sensory monotony. In environments with restricted novelty, limited sensory diversity may therefore contribute to the reduced energy intake frequently reported in isolated and confined settings. The presence of firmer components in many meals indicates that textural variation is feasible within the existing supply system. Adjusting color contrasts and texture combinations may therefore represent low-resource strategies to support food acceptance and eating motivation in constrained environments.

Third, comparison between planned menus and photographed meals revealed limited agreement, with meals frequently differing in preparation details or accompanying components. These discrepancies likely reflect multiple overlapping factors, including logistical constraints such as stock depletion, operational adaptations such as the use of leftovers, cook-specific decisions, and crew preferences, though the relative contribution of each factor could not be determined as deviations were not systematically documented. Certain components such as desserts may additionally be underrepresented, as they were prepared less frequently and more likely documented when newly introduced, based on reports from the study physicians. Consequently, planned menus only partially reflected what was actually served. This discrepancy has methodological implications, as studies relying exclusively on menu plans may capture intended rather than actual food environments. Similar discrepancies between planned menus and foods actually served have been reported in institutional food-service settings, including childcare facilities (57). Image-based documentation offers a complementary approach by enabling direct observation of foods served under real-world conditions (26), and combined methods may provide the most comprehensive characterization of food environments in isolated and operational settings where flexibility is required.

### 4.1 Strengths and limitations

A key strength of this study is the image-based assessment of the experienced food environment as documented by the study physicians under Antarctic ICE conditions. This approach allowed objective evaluation of meal composition, variety, and sensory properties while avoiding recall bias. Data from three overwintering campaigns enabled exploration of inter-mission variability. Independent ratings and low participant burden further support the robustness and feasibility of this approach. Because image-based documentation can be implemented with minimal logistical demands, this approach may also be applicable to other isolated and operational settings, including spaceflight analogs and future long-duration missions.

Limitations include variable image quality and incomplete coverage across campaigns, as documentation was conducted by different physicians without full standardization, limiting inter-mission comparability. Furthermore, as food group, color, and texture classifications were based on subjective visual assessment by trained human raters rather than automated or instrument-based measurement, some degree of rater-dependent variability cannot be excluded, despite the generally acceptable interrater reliability observed. Documentation focused on lunch and dinner, capturing the self-selected plates of the study physicians within the shared meal setting. These findings therefore describe this specific segment of the food environment rather than overall energy intake or the full range of foods offered to or consumed by the entire crew. Although drawn from the same centrally prepared menu, the photographed plates likely represent only an approximation of the broader food environment, and selection bias cannot be excluded given that meals were self-selected. Because the images capture what was offered rather than what was consumed, portion sizes, hidden ingredients, and nutrient composition cannot be reliably derived from the photographs. The method is therefore suited to identifying general patterns in meal structure but less appropriate for detailed nutrient evaluation. Color-related findings are subject to additional constraints, as color assessment relied on one or more characteristic pixels per food item rather than whole-image segmentation, and no color calibration or white-balance normalization was feasible; absolute hue values thus reflect the specific capture conditions rather than standardized color measurements, warranting particular caution in between-campaign comparisons. The use of broad color categories rather than fine-grained hue distinctions may partially mitigate this limitation. Texture classifications were based on visual approximation of IDDSI levels from photographs rather than confirmed through formal physical IDDSI testing, and should therefore be interpreted as descriptive approximations rather than validated texture measurements. Nonetheless, the dataset offers a rare operational perspective on food environments in ICE settings.

## 5 Conclusion and outlook

This image-based analysis provides a unique, operationally grounded perspective on the food environment during Antarctic overwintering, based on self-selected lunch and dinner plates of the research physicians. Meal provision appeared structured and showed limited variation across campaigns on descriptive assessment, with the proportional contribution of vegetables remaining relatively stable during overwintering. Sensory variety showed some limitations, suggesting moderate but realistic potential for improvement. Planned menus only partly reflected foods actually served, highlighting the value of direct documentation. Standardized and potentially automated image-based methods may therefore represent a useful complementary tool for monitoring and optimizing food provision in ICE settings. Future developments could involve the integration of AI-assisted image analysis to support automated food recognition and more scalable data processing. The structured image dataset generated in this study may provide a useful basis for developing and testing such approaches, potentially improving the objectivity and efficiency of food-environment monitoring. Overall, these findings support the feasibility of maintaining a structured dietary environment under prolonged isolation and inform evidence-based planning for future missions.

## Data Availability

The original contributions presented in the study are included in the article/Supplementary material, further inquiries can be directed to the corresponding author.
