# Supplementary material for: Dietary supply and reporting reliability during Antarctic overwintering under extreme isolation: a food image-based analysis
Source: Front Nutr. 2026 Aug 19;13:1855383. doi: 10.3389/fnut.2026.1855383 (PMC13533749; doi:10.3389/fnut.2026.1855383)
Supplement: Supplementary file 1 [file Data_Sheet_1.pdf]

Dietary Supply and Reporting Reliability during Antarctic Over-wintering under Extreme Isolation: A Food Image-Based Analysis

Supplementary Material

Verena Steinhauser[1†], Bea Klos[1†], Rebecca Föhr[1], Sophia Wolf[1], Eva Dichiser[1], Julia Low[2], Christine Brombach[3], Nadja Albertsen[4–6], Stijn Thoolen[4,7], Hannes Hagson[4], Paul Enck[1], Isabelle Mack[1]*

[1]Internal Medicine VI, Psychosomatic Medicine and Psychotherapy, University Hospital Tübingen, Tübingen, Germany

[2]Sensory and Consumer Science Research Group, School of Science, RMIT University, Bundoora West Campus, Bundoora, Victoria, Australia

[3]Research Group for Food Perception, ZHAW School of Life Sciences and Facility Management, Waedenswil, Switzerland

[4]French Polar Institute Paul-Emile Victor, Brest, France - sponsored by the European Space Agency

[5]Aarhus University, Denmark

[6]Aarhus University Hospital, Denmark

[7]Department of Psychiatry, Massachusetts General Hospital, Harvard Medical School, Boston, MA, USA

[†]These authors contributed equally to this work and share first authorship.

***Corresponding author:** Prof. Dr. Isabelle Mack, isabelle.mack@uni-tuebingen.de

A 08 February 2020 | Lunch, main dish

Menu plan: Beetroots, corn, chorizo; ebly (wheat) with vegetables (carrots, celery, shallot, basil); eggplant, grilled

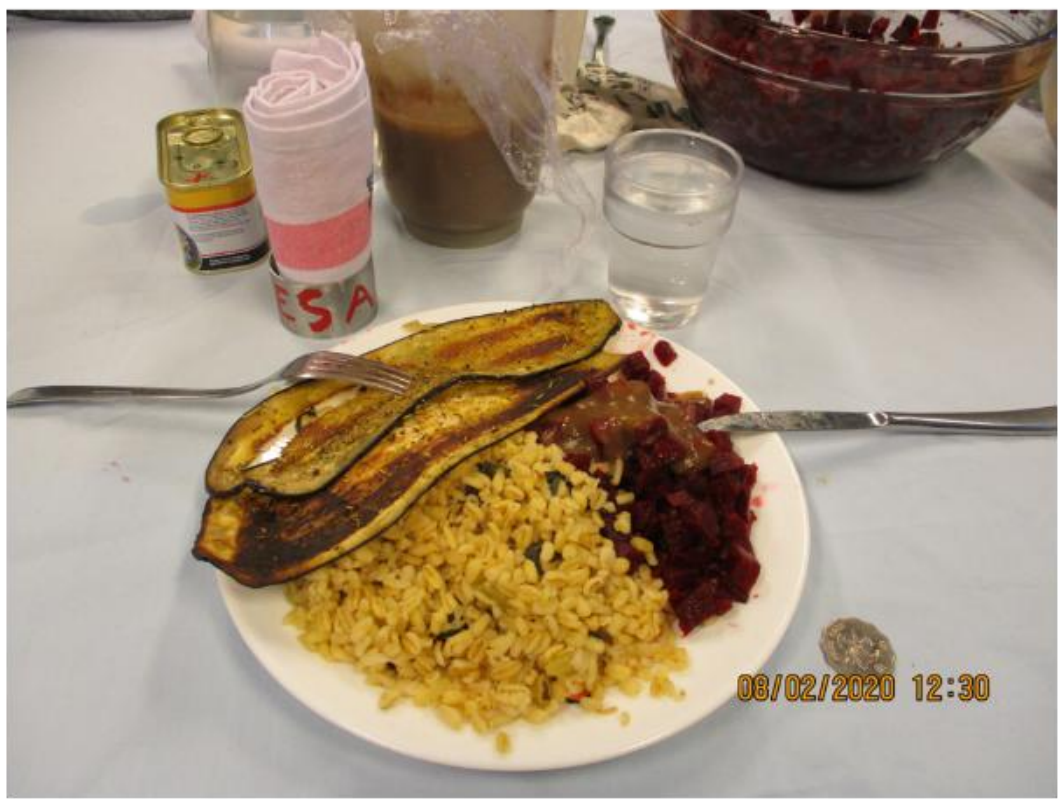

1 **Cereals & cereal products**
(50% of plate, HSL: 44-0.63-0.55 (yellow), IDDSI Level 5)

2 **Vegetables**
(45% of plate, HSL: 6-0.62-0.12 (red), HSL: 38-0.65-0.38 (yellow), HSL: 41-0.68-0.51 (yellow), IDDSI Level 5)

3 **Soups & Sauces**
(5% of plate, HSL: 29-0.40-0.42 (orange), IDDSI Level 3)

B 03 March 2020 | Lunch, main dish

Menu plan: Pasta with tomato sauce (carrots, onions, bacon / egg for vegetarians)

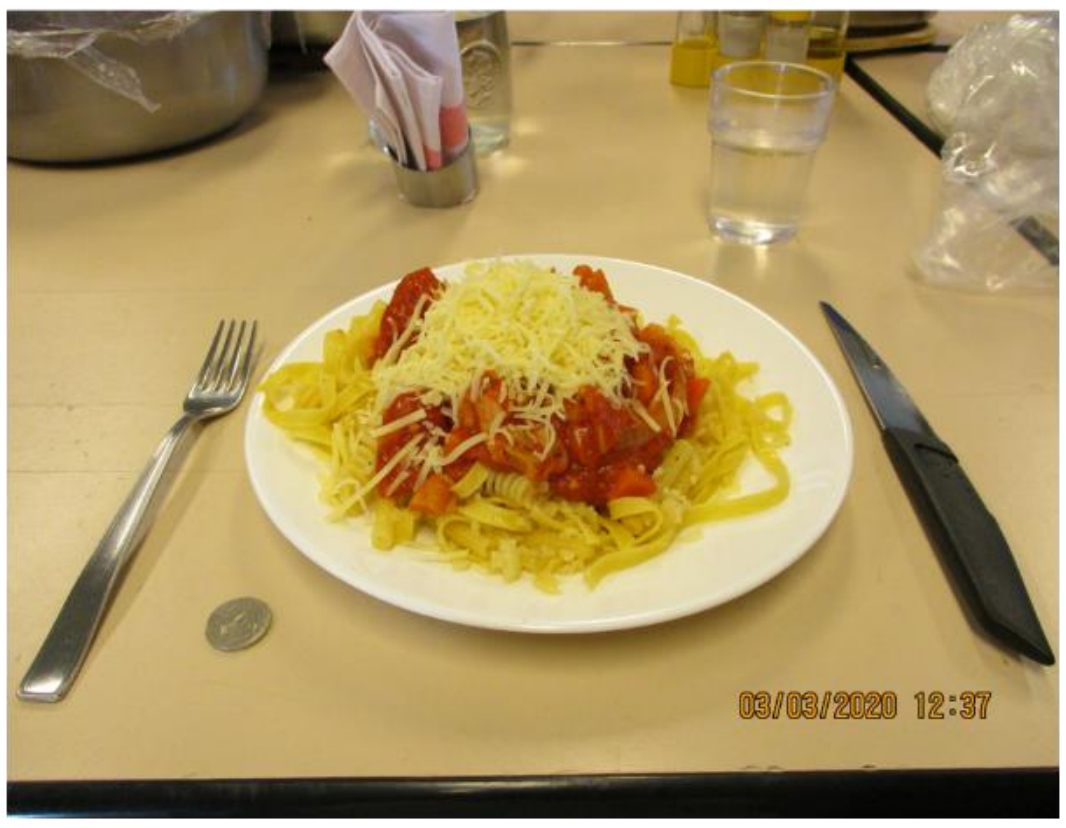

1 **Cereals & cereal products**
(60% of plate, HSL: 50-0.73-0.47 (yellow), IDDSI Level 6)

2 **Vegetables**
(20% of plate, HSL: 21-0.97-0.28 (orange), IDDSI Level 5)

3 **Soups & Sauces**
(10% of plate, HSL: 20-0.74-0.34 (orange), IDDSI Level 3)

4 **Milk & dairy products**
(10% of plate, HSL: 51-0.63-0.59 (yellow), IDDSI Level 5)

C 01 April 2020 | Lunch, main dish

Menu plan: Polenta and vegetables; tenderloin of pork

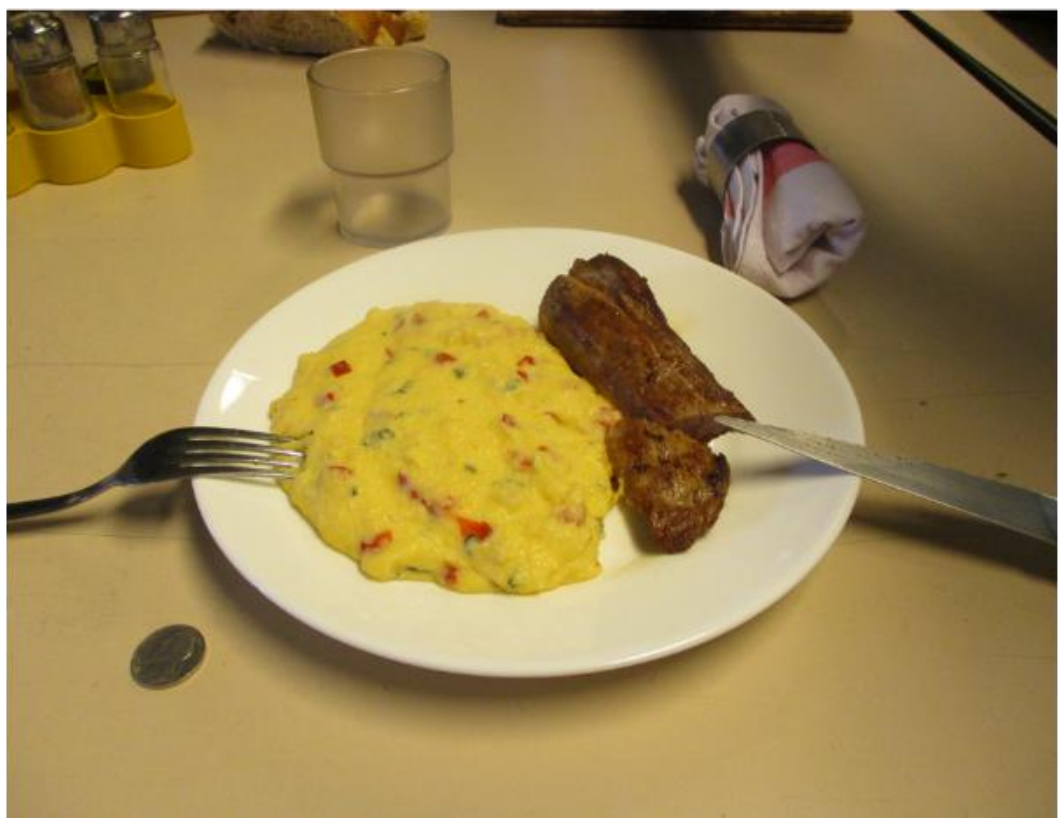

1 **Cereals & cereal products**
(50% of plate, HSL: 48-0.67.0.54 (yellow), IDDSI Level 4)

2 **Vegetables**
(5% of plate, HSL: 39-0.59.0.49 (yellow), HSL: 51-0.52-0.41 (yellow), IDDSI Level 5)

3 **Meat & meat products**
(45% of plate, HSL: 34-0.77-0-25 (orange), IDDSI Level 6)

Supplementary Figure S1. Representative example images illustrating the applied classification criteria for food group assignment, color coding, and texture classification.

Three representative meal photographs from the 2019/2020 campaign are shown for illustrative purposes: (A) 08 February 2020, Lunch – Main dish. (B) 03 March 2020, Lunch – Main dish. (C) 01 April 2020, Lunch– Main dish. For each meal, the planned menu is indicated. Food group categories were assigned based on a scheme adapted from the European Prospective Investigation into Cancer and Nutrition (EPIC) framework; proportional contributions to total

plate content were estimated visually by trained raters. Color characteristics were assessed using HSL (Hue–Saturation–Lightness) values sampled via a digital color picker integrated in the analysis tool; dominant hues were assigned to predefined color categories based on hue angle ranges and are indicated in parentheses. Texture was classified according to the IDDSI framework (Steele et al., 2015); levels 3–7 reflect a continuum from liquidised to regular textures.

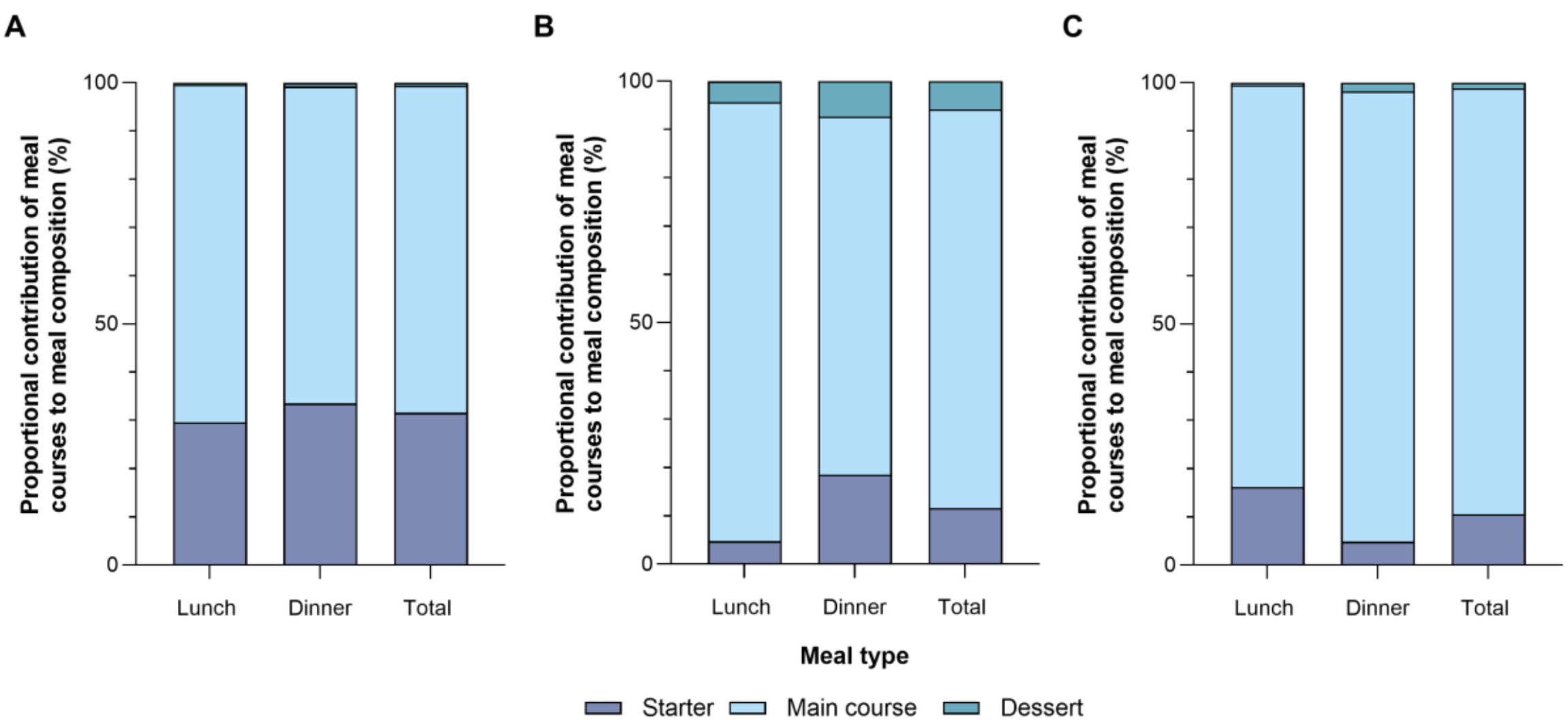

Supplementary Figure S2. Proportional distribution of meal courses across overwintering campaigns.

A Overwintering campaign 2018/2019, **B** overwintering campaign 2019/2020, and **C** overwintering campaign 2021/2022. Bars show the mean proportional contribution (%) of dessert, main course, and starter to the whole meal, presented separately for lunch, dinner, and combined totals. Proportions are expressed relative to the total meal composition per recorded meal.

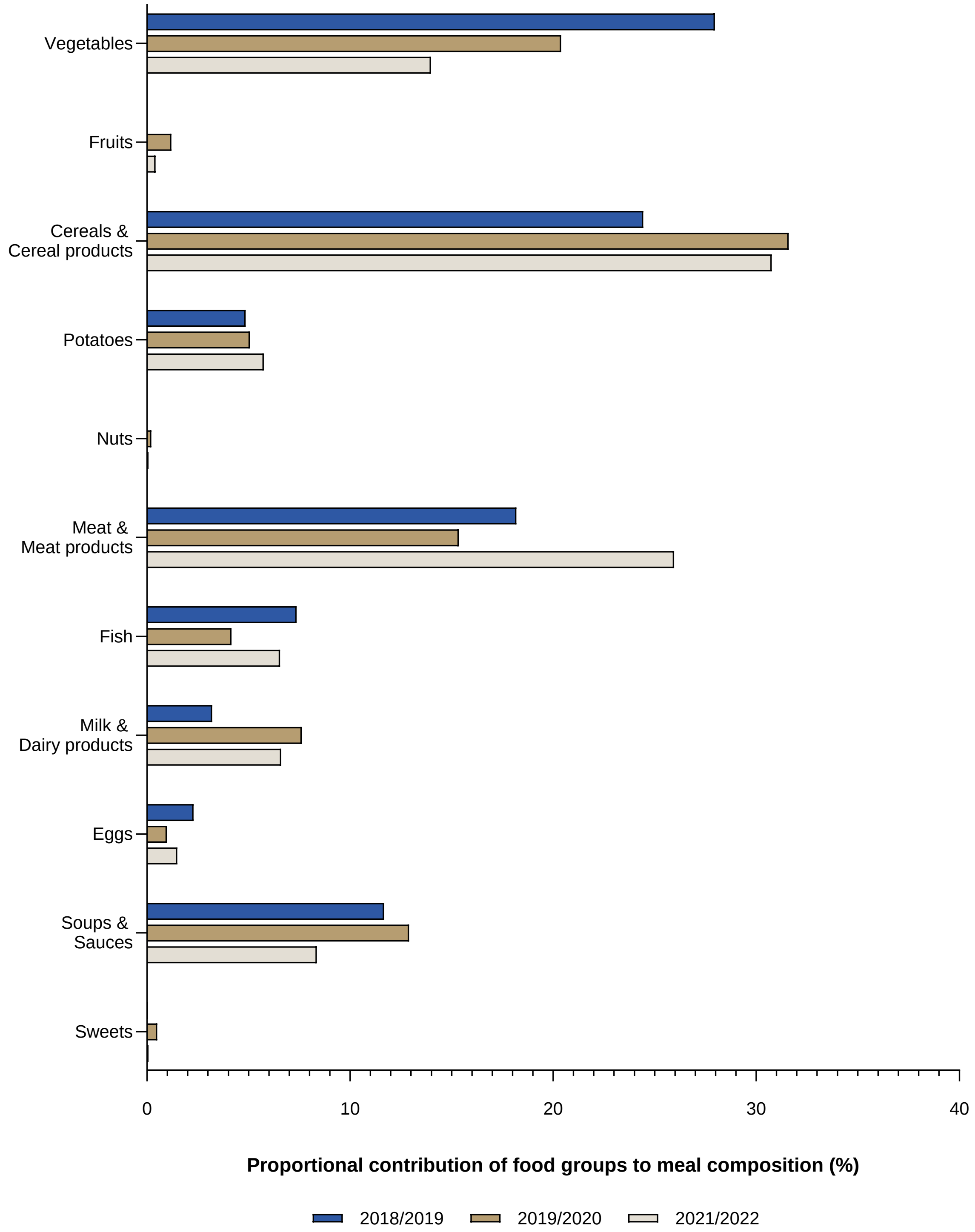

Supplementary Figure S3. Proportional contribution of food groups to meals across overwintering campaigns.

Mean proportional contribution (%) of individual food groups to the whole meal is shown for overwintering campaigns 2018/2019 (blue), 2019/2020 (tan), and 2021/2022 (light gray). Bars represent campaign-specific mean percentages, allowing comparison of the relative contribution of food groups to overall meal composition across overwintering campaigns.

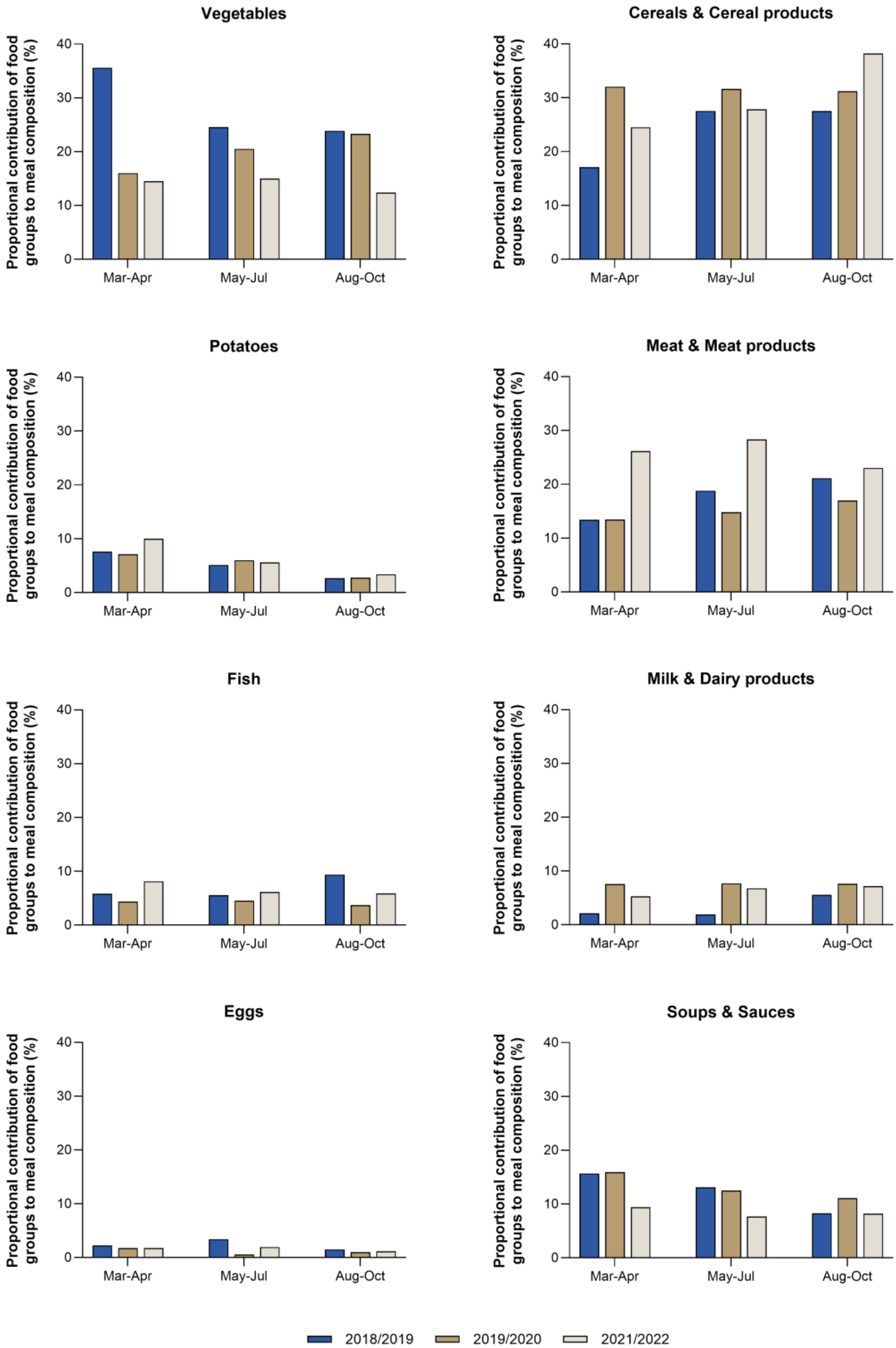

Supplementary Figure S4. Proportional contribution of food groups to meals across time periods and overwintering campaigns.

Mean proportional contribution (%) of individual food groups to the whole meal is shown by time period for overwintering campaigns 2018/2019 (blue), 2019/2020 (tan), and 2021/2022 (light gray). Bars represent mean percentages for early (March–April), mid (May–July), and late (August–October) phases of the overwintering mission.

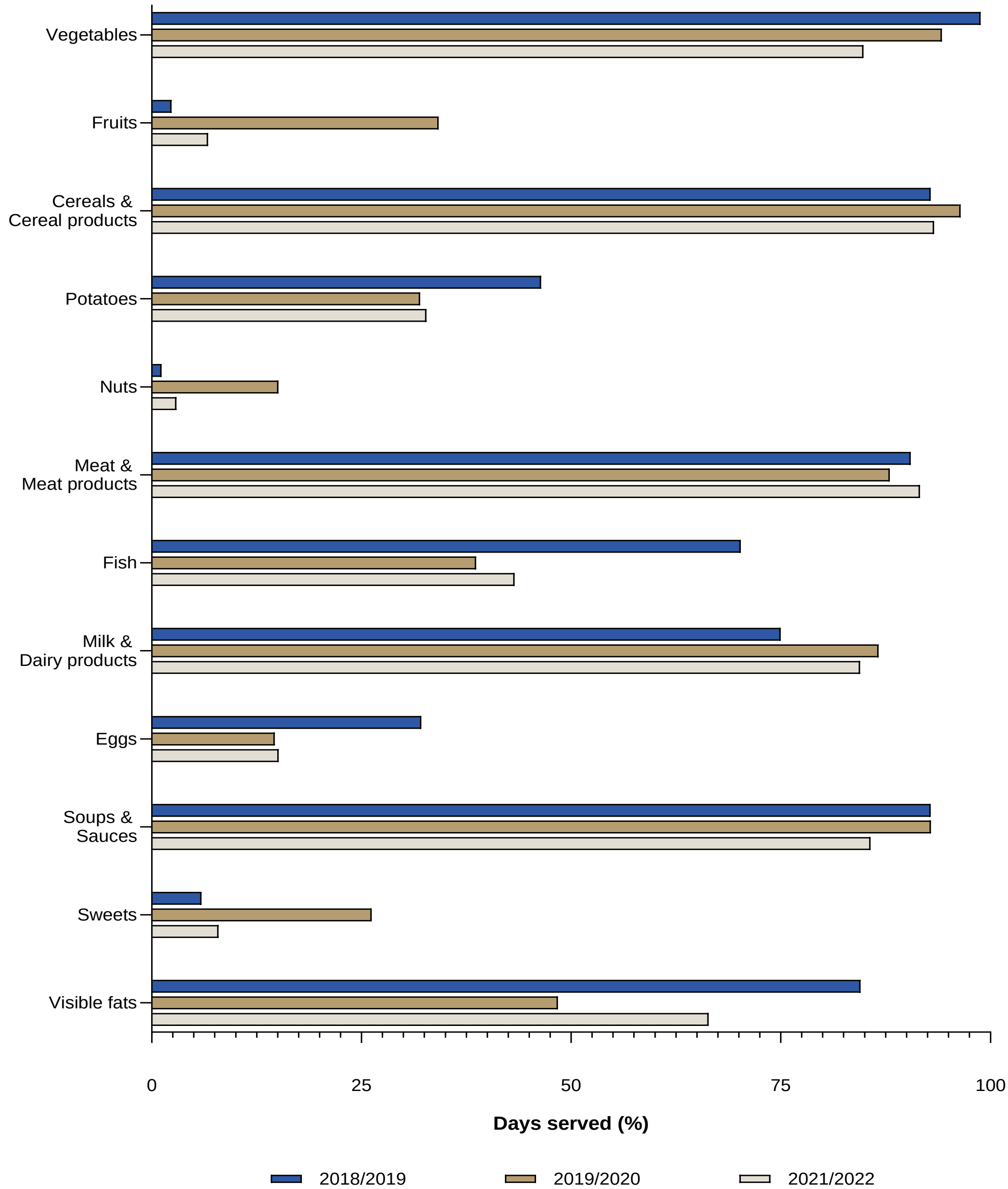

Supplementary Figure S5. Frequency of food group provision across overwintering campaigns.

Mean frequency (%) of food group provision is shown for overwintering campaigns 2018/2019 (blue), 2019/2020 (tan), and 2021/2022 (light gray). Bars represent the mean percentage of recorded days on which each food group was served. Frequencies are expressed relative to the total number of recorded days per campaign.

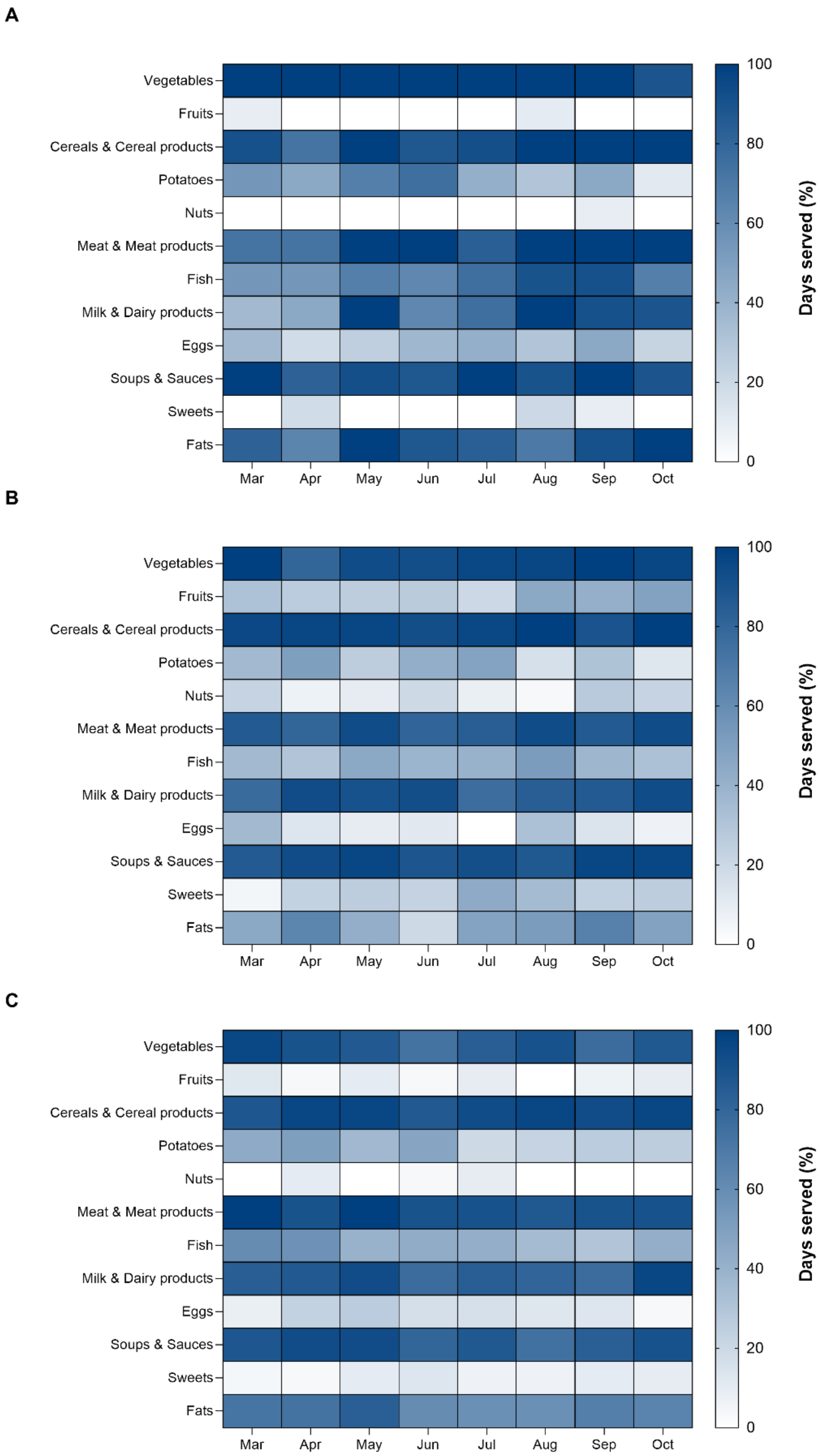

Supplementary Figure S6. Monthly frequency of food group provision across overwintering campaigns.

A Overwintering campaign 2018/2019, **B** overwintering campaign 2019/2020, and **C** overwintering campaign 2021/2022. Heatmaps show the mean percentage of recorded days on which each food group was served, summarized by month. Color intensity reflects serving frequency, with darker shades indicating higher percentages and lighter shades lower percentages.

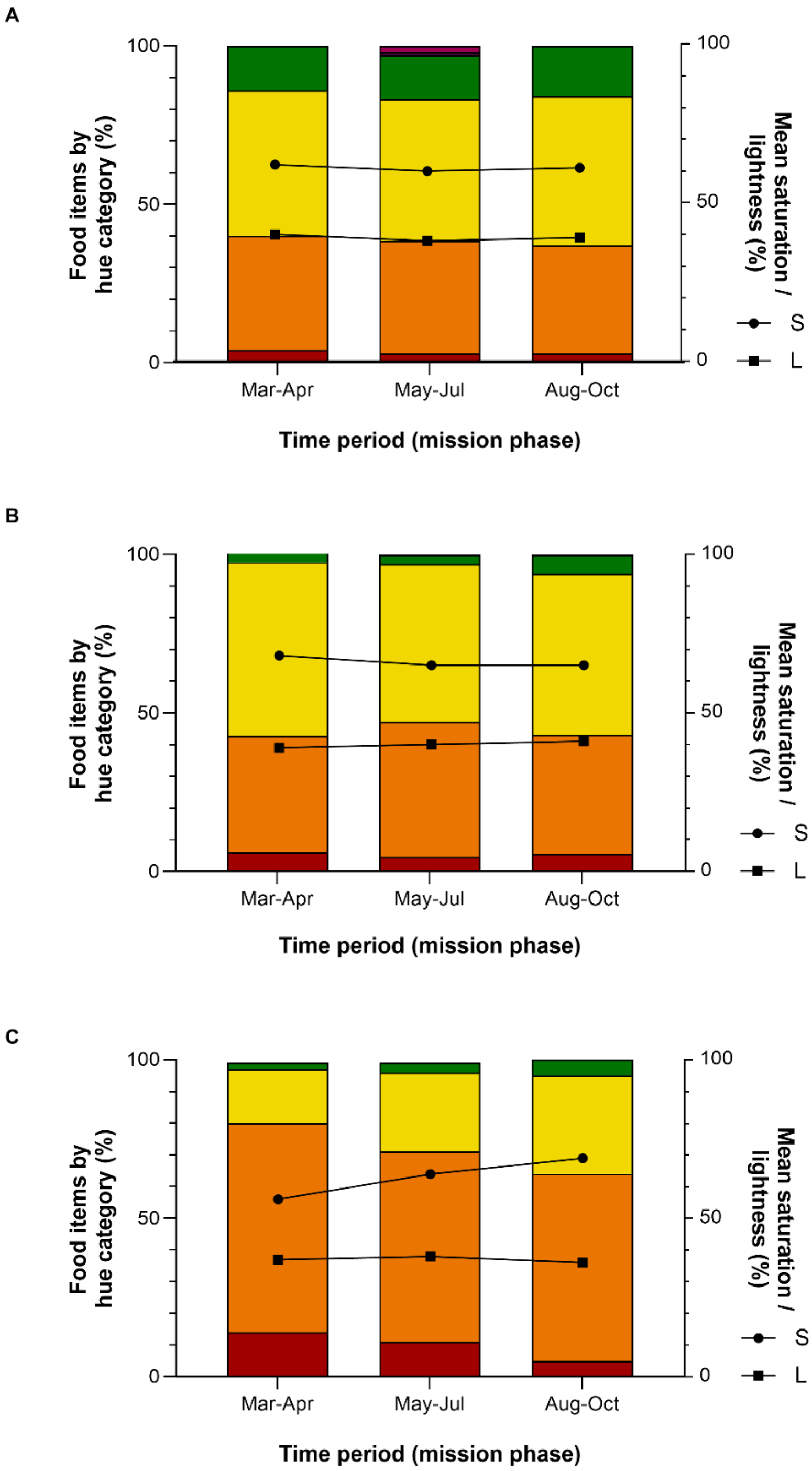

Supplementary Figure S7. Color classification by time period across overwintering campaigns.

A Overwintering campaign 2018/2019, **B** Overwintering campaign 2019/2020, and **C** Overwintering campaign 2021/2022. Time periods represent phases of the overwintering mission (early (March-April), mid (May-July), and late (August-October), allowing comparison of visual food characteristics across the course of confinement. Stacked bars show the mean percentage of documented food items assigned to each color category, based on the hue value of characteristic pixels selected per food item and converted from hexadecimal to HSL values. Color classification was performed in the HSL color space using predefined Hue (H) angle ranges: reds (0–15° and 345–360°), oranges (15–40°), yellows (40–55°), greens (55–135°), magentas

(285–315°), and magenta-reds (315–345°). Overlaid symbols indicate mean Saturation (S; circles) and Lightness (L; squares) values by time period. Saturation reflects chromatic intensity or color purity (lower values indicating more desaturated or grayish tones, higher values more vivid colors), whereas Lightness represents perceived brightness from dark to light. Together, S and L complement hue-based classification by further characterizing the visual appearance of foods.

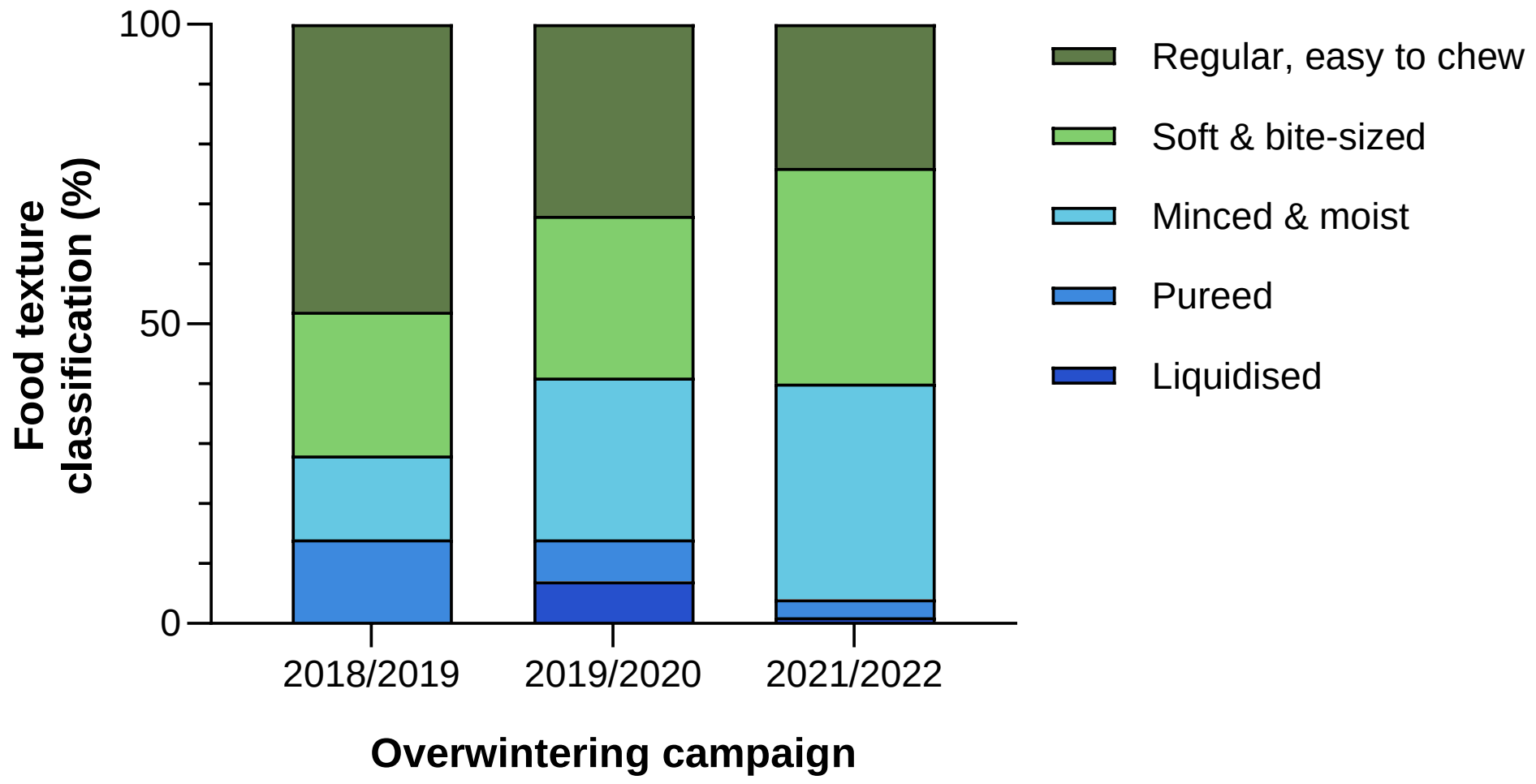

Supplementary Figure S8. Distribution of food textures across overwintering campaigns.

Mean percentages of food textures classified according to the International Dysphagia Diet Standardisation Initiative (IDDSI) framework are shown for overwintering campaigns 2018/2019, 2019/2020, and 2021/2022. Bars represent campaign-specific percentages, allowing comparison of texture distribution across overwintering campaigns.
